# Genetic diversity of *Ascaris* spp. infecting humans and pigs in distinct Brazilian regions, as revealed by mitochondrial DNA

**DOI:** 10.1371/journal.pone.0218867

**Published:** 2019-06-24

**Authors:** Kerla J. L. Monteiro, Deiviane A. Calegar, Jessica P. Santos, Polyanna A. A. Bacelar, Beatriz Coronato-Nunes, Elis Regina C. Reis, Márcio N. Boia, Filipe A. Carvalho-Costa, Lauren H. Jaeger

**Affiliations:** 1 Laboratório de Epidemiologia e Sistemática Molecular, Instituto Oswaldo Cruz, Fundação Oswaldo Cruz, Rio de Janeiro, Rio de Janeiro, Brazil; 2 Escritório Técnico Regional, Fundação Oswaldo Cruz Piauí, Teresina, Piauí, Brazil; 3 Laboratório de Biologia e Parasitologia de Mamíferos Silvestres Reservatórios, Instituto Oswaldo Cruz, Fundação Oswaldo Cruz, Rio de Janeiro, Rio de Janeiro,Brazil; National Cheng Kung University, TAIWAN

## Abstract

In this study, we assessed the genetic diversity of *Ascaris lumbricoides* / *Ascaris suum* circulating in humans and pigs, exploring potential zoonotic cycles in endemic areas in Brazil. We carried out cross-sectional surveys in four municipalities: Santa Isabel do Rio Negro (SIRN-AM) (n = 328); Nossa Senhora de Nazaré (NSN-PI) and Teresina (TER-PI) (n = 605 and n = 297, respectively); and Cachoeiras de Macacu (CAM-RJ) (n = 543). We also studied 61 fecal samples/adult worms obtained from pigs (n = 53 in NSN-PI and n = 8 in TER-PI). A ~450 bp fragment of the *Ascaris* cytochrome c oxidase subunit 1 (*cox*1) and ~400 bp of the NADH dehydrogenase subunit 1 (*nad*1) were amplified and sequenced. Maximum-likelihood (ML) tree and Median-joining (MJ) haplotype network analyses were performed. We also performed scanning electron micrographs of adult specimens. Positivity rates were 93/328 (28.4%) in SIRN-AM, 6/297 (2.0%) in TER-PI, 0/605 (0%) in NSN-PI, and 6/543 (1.1%) in CAM-RJ. In NSN-PI it reached 11/53 (20.7%) in pigs. The MJ network based on *cox*1 locus (383 bp) revealed three main clusters, one centered around haplotypes H01/H28/H32 and the other around H07/H11. The *cox*1 haplotypes had a heterogeneous distribution, showing no pattern by geographic region, and high haplotype diversity. The ML trees based on *cox*1 and *nad*1 loci showed a similar topology with each other, and with the haplotype networks. Three distinct clusters were observed. Sequences of *cox*1 and *nad*1 from humans and animals were distributed throughout the tree and it was not possible to differentiate specimens of human and swine origin. *Ascaris* populations obtained from humans and swine in different Brazilian regions are not discriminable through the genetic markers used, which indicates the potential for zoonotic transmission and the need for better control of these infections in swine herds, mainly when created in a peridomestic environment.

## Introduction

The soil-transmitted helminthes (STH) *Ascaris lumbricoides* and *Ascaris suum* are parasites of great importance to human and swine health, respectively [1,2]. In humans, *A*. *lumbricoides* can cause potentially serious and fatal disease when high parasitic loads are present. In these cases, ascariasis can be associated with intestinal obstruction and perforation, in addition to the migration of adult worms to the bile duct and other organs [3,4]. Chronic infections by roundworms may also be associated with delayed cognitive and physical development in children [5,6]. Infection by roundworms with high parasitic load is dependent on constant reinfection and continuous exposure to the environment contaminated with fecal matter [7]. Ascariasis has been successfully controlled in developed countries through sanitation and the adequate disposal of human feces. However, in many areas in developing countries, the disease persists as an important public health problem, with a high prevalence rate and infection intensity.

Ascariasis control strategies are strongly based on chemoprophylaxis campaigns (MDA [mass drug administration]), mainly in Africa, Asia, and Latin America, with a single 400 mg dose of albendazole [8–10]. This strategy aims to reduce prevalence, control parasitic burden, and reduce transmission potential. In Brazil, the Ministry of Health has implemented these actions on a more regular basis since 2012 with the National Soil-transmitted Helminthiases Campaign [11].

Classically considered an anthroponotic disease, there is evidence that ascariasis can cross the host species barrier (humans and pigs), circulating at the human-animal interface [12–14]. Nevertheless, the importance of potential zoonotic transmission and cross-infections in disease production and the maintenance of endemicity in specific communities needs to be better understood. In the case of infection in swineherds, the disease is controlled by the regular administration of ivermectin or benzimidazole compounds, such as fenbendazole [15].

The hypothesis that ascariasis is potentially zoonotic and produces cross-infections is based on the close similarity between the parasites *A*. *lumbricoides* and *A*. *suum*. Morphologically, there are very few differences, these being restricted to details detectable by electron microscopy in the shape of the lips and size of the teeth, as evaluated and reviewed by Ansel and Thibaut [16]. From the perspective of molecular evolutionary genetics, there is high homology in the nucleotide and amino acid sequences of the two species, suggesting that *A*. *lumbricoides* and *A*. *suum* are a single species [17]. The evidence that humans and pigs can be infected with both nematodes supports the hypothesis that *A*. *lumbricoides* and *A*. *suum* actually represent a single zoonotic species [18–23].

In this study, we assessed the genetic diversity of *Ascaris lumbricoides* / *Ascaris suum* circulating in humans and pigs, exploring potential zoonotic cycles in endemic areas in Brazil.

## Material and methods

### Study design, population, and sampling strategy

We evaluated the presence of *A*. *lumbricoides/A*. *suum* in 1,773 humans and 61 pigs in three Brazilian regions (Rio de Janeiro [RJ], Piauí [PI], and Amazonas [AM] states). In order to assess the presence of roundworms, we carried out cross-sectional surveys in four municipalities: Santa Isabel do Rio Negro (SIRN-AM); Nossa Senhora de Nazaré (NSN-PI) and Teresina (TER-PI); and Cachoeiras de Macacu (CAM-RJ). These regions are extremely diverse in climatic, physiogeographic, and sociodemographic aspects, as shown in Table 1. In the states of AM and RJ, the study only involved humans. In the state of PI, humans and pigs were studied (Table 1). In all the studied localities, residents infected with helminths or pathogenic protozoa were treated with specific antiparasitic drugs, including albendazole, metronidazole, and secnidazole. In the four municipalities studied, members of the research team visited households and delivered bottles for fecal collection. A questionnaire was administered at the same time to obtain sociodemographic and sanitation data. In the state of PI, the study involved humans and pigs. The adult worms from pigs were obtained from necropsies, and swine feces were collected after spontaneous evacuation. The adult worm from human participants was obtained by spontaneous elimination after treatment with a single 400 mg dose of albendazole (chemo-expelled).

**Table 1:** Characteristics of Brazilian localities and samples included in this study.

Characteristic	Site of collection			
	Cachoeiras de Macacu, RJ	Teresina, PI	Nossa Senhora de Nazaré, PI	Santa Isabel do Rio Negro, AM
**Kind of seattlement**	Urban	Rural	Rural	Urban
**Year of collection**	2018	2017	2016-2015-2014	2011
**Region**	Southeast	Northeast	Northeast	North
**Climate**	Tropical	Tropical, semiarid	Tropical, semiarid	Equatorial
**Annual pluviosity**	1307 mm	1349 mm	1421 mm	2497 mm
**Temperature range average**	23.1°C	27.6°C	26.8°C	26.8°C
**IDHM**	0.752	0.751	0.594	0.479
**Human fecal samples collected**	543	297	605	328
**Human fecal samples positive for *Ascaris* sp.**	6	6	0	93
**Adult worms obtained from humans**	0	1	0	0
***Ascaris* sp. sequences obtained from humans**	4	6	0	74
**Pig fecal samples collected**	0	8	53	0
**Pig fecal samples positive for *Ascaris* sp.**	-	3	11	-
**Adult worms obtained from pigs**	0	2	0	0
***Ascaris* sp. sequences obtained from pigs**	-	2	2	-

MHDI: Mean human development index. RJ: Rio de Janeiro state; PI: Piauí state; AM: Amazonas state.

### Laboratory procedures and detection of *Ascaris lumbricoides* / *Ascaris suum* in fecal samples

Fresh fecal samples were processed through the Ritchie and Kato-Katz techniques. Moreover, samples obtained from pigs were also examined by sucrose flotation. *A*. *lumbricoides/A*. *suum* positive samples (original fecal samples) were cryopreserved and transported to the Oswaldo Cruz Foundation, in Rio de Janeiro. Spontaneous sedimentation was performed before the molecular analysis. Adult worms were preserved in 70% ethanol until processing and then washed twice with sterile saline for two hours. Forty mg of the adult worms was cut with sterile scalpel, macerated with mini-pistil, and digested with 20 mg/mL of Proteinase K (Invitrogen, Waltham, MA, USA).

### Electron microscopy of adult worms

For the scanning electron microscopy, the specimens were fixed in 2.5% glutaraldehyde, and post-fixed in 1% osmium tetroxide, both in 0.1 M sodium cacodylate buffer at pH 7.2. They were washed in the buffer and then dehydrated in an increasing ethanol series. After being critical-point dried using super-dry CO_2_ in a Balzers device, the material was mounted on metal supports, coated with gold, and observed using a JEOL JSM 6390LV scanning electron microscope (JEOL Ltd., Akishima, Tokyo, Japan) in the Rudolph Barth Electron Microscopy Platform, Instituto Oswaldo Cruz, Fiocruz.

### Ethics

The study was approved by the Research Ethics Committee (license CAAE 12125713.5.0000.5248) and the Ethics Committee for Animal Use (license LW-21/13 [P-4/13.3]) of the Oswaldo Cruz Institute, Fiocruz. All subjects provided written informed consent and the parent or legal guardian of all children included in this study provided written informed consent on their behalf.

### DNA extraction, polymerase chain reaction, and sequencing

Parasitic DNA was obtained from three different sources: i) eggs obtained by spontaneous sedimentation (400 μL of fecal sediment); ii) eggs obtained by Kato-Katz thick smears, as described by [24]; and iii) adult worms obtained by the necropsy of slaughtered pigs or chemo-expulsion in humans. DNA extraction was performed using a DNeasy Blood & Tissue Kit (Qiagen, Hilden, Germany) according to the manufacturer's recommendations. The polymerase chain reaction (PCR) was performed using a Platinum Taq DNA Polymerase kit (Invitrogen, Waltham, MA, USA), with 50 μL of final volume. *A*. *lumbricoides/A*. *suum* mitochondrial targets cytochrome c oxidase subunit 1 (*cox*1, ~450bp) and NADH dehydrogenase subunit 1 (*nad*1, ~400bp) were amplified [25]. PCR conditions were as described by the authors. The amplicons were purified using Illustra GFX PCR DNA and Gel Band Purification Kit (GE HealthCare, Pittsburgh, PA, USA) and were sequenced using a BigDye Terminator v.3.1 Cycle Sequencing Ready Reaction kit (Applied Biosystems, Foster City, CA, USA) in an ABI3730 Automated DNA Sequencer (Applied Biosystems). *Ascaris lumbricoides*/*Ascaris suum* nucleotide sequences were edited with Bioedit v.7.2.5 [26] software. The GenBank accession numbers were MK143378-MK143391 and MK160501-MK160574. The *cox*1 sequences of SIRN-AM were published in [24], accession numbers MH674438-MH674442 and MH800218-MH800286.

### Phylogenetic inferences, genetic diversity, and haplotype network

The Basic Local Alignment Search Tool (BLAST, NCBI https://blast.ncbi.nlm.nih.gov/Blast.cgi) was used to check similarity with *Ascaris* sequences. Orthologous sequences representing the diversity of *Ascaris* spp. were retrieved from GenBank (NCBI, https://www.ncbi.nlm.nih.gov/nucleotide/) and added to the analyses. *Ascaris* spp. nucleotide sequences were aligned using the multiple sequence alignment ClustalW [27] available in the BioEdit v.7.2.5 [26] software. The substitution model for the *cox*1 and *nad*1 dataset was chosen using Bayesian Information Criterion (BIC) in Molecular Evolutionary Genetics Analysis (MEGA) v.7.0.20 software [28]. The lower BIC score was the Hasegawa-Kishino-Yano (HKY) model with non-uniformity of evolutionary rates among sites (+G). The Maximum Likelihood (ML) method was implemented in MEGA v.7.0.20 software [28], using the HKY+G model. Branch support was provided by bootstrapping with 1,000 replications.

DNA Sequence Polymorphism (DNASP) v.6 [29] was used to prepare an input file. A Median-joining (MJ) haplotype network was constructed in Network v.5.0.0.3 software [30] (Fluxus Technology Ltd., www.fluxusengineering.com). In order to evaluate the intraspecific genetic diversity of Brazilian *Ascaris* populations, diversity indices were determined for each population pair using Pairwise Distance in ARLEQUIN v.3.5.2.2 software (http://cmpg.unibe.ch/software/arlequin35/) [31]. Orthologous sequences representing the Brazilian diversity of *Ascaris* spp. were retrieved from [20] (n = 3, from Guapimirim/RJ [GUA-RJ]) and [21] (n = 7, from SIRN-AM 2008, MG, and from Rio de Janeiro-RJ). The map was created using Terraview v.4.2.2 software (www.dpi.inpe.br/terraview).

## Results

As presented in Table 1, *Ascaris lumbricoides / Ascaris suum*-positive samples were found in all localities studied. Eighty-eight *cox*1 *Ascaris* sequences (424 pb, 5 short sequences removed) and 74 *nad*1 *Ascaris* sequences (381 pb, 6 short sequences removed) were obtained. Alignments were performed with our new sequences (83 *cox*1 and 68 *nad*1 sequences) and *Ascaris* spp. reference sequences (169 *cox*1 and 53 *nad*1 sequences) from GenBank (S1 Table).

**Fig 1:**
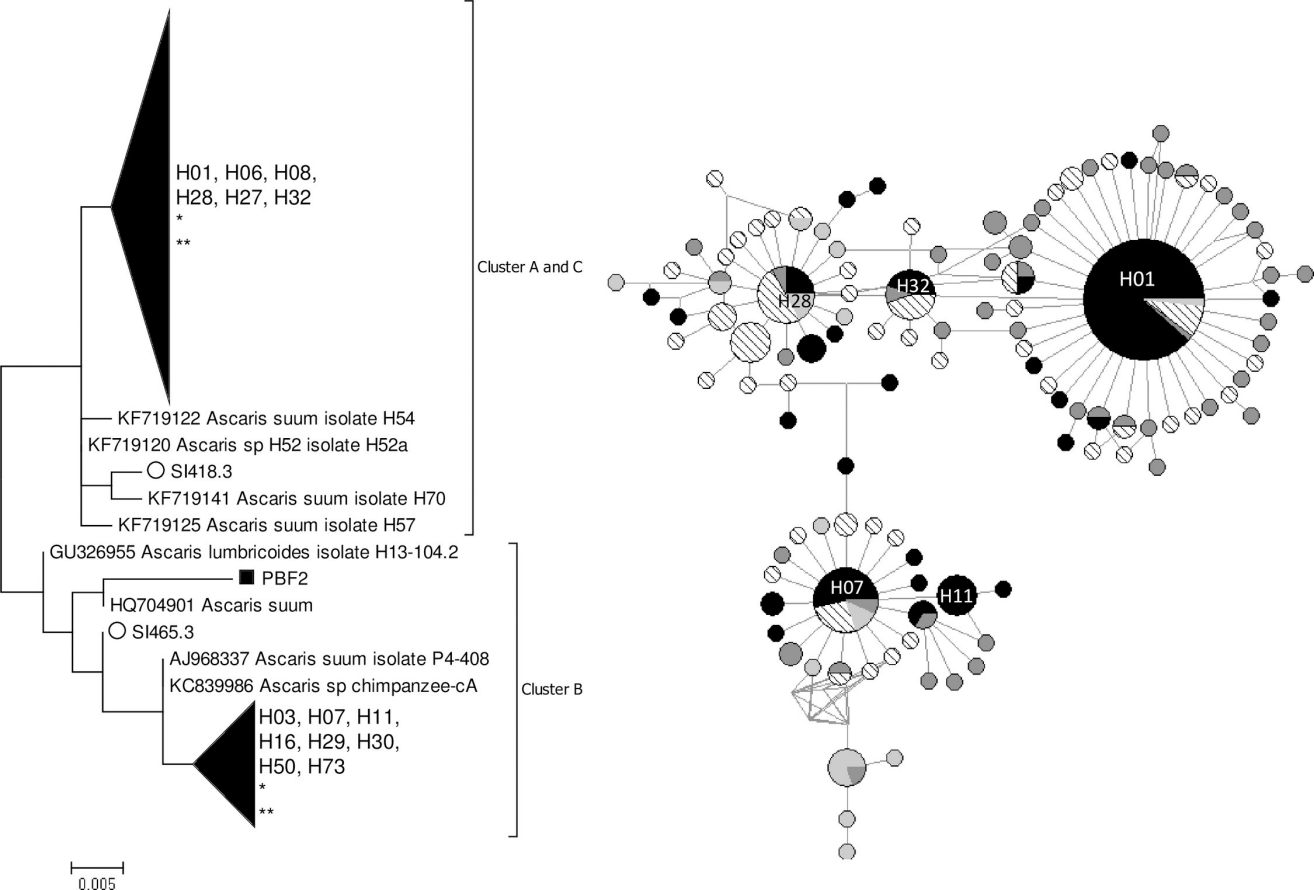
ML tree and MJ network of 383 bp *cox*1 locus of *Ascaris* spp. (252 sequences). The colors of the circles indicate the isolation continent: black: America; dark gray: Africa; diagonal: Asia; and light gray: Europe. The area of the circle is proportional to the sequence number. *: pig origin samples. **: non-human primate sample. Only bootstrap values ≥70% are reported.

The ML trees based on *cox*1 (Fig 1) and *nad*1 (S1 Fig) loci showed a similar topology with each other, and with the haplotype networks. Three distinct clusters were observed, corresponding to the A-C clusters described by [32]. Sequences of *cox*1 and *nad*1 from humans and animals were distributed throughout the tree and it was not possible to differentiate specimens of human and swine origin.

**Fig 2:**
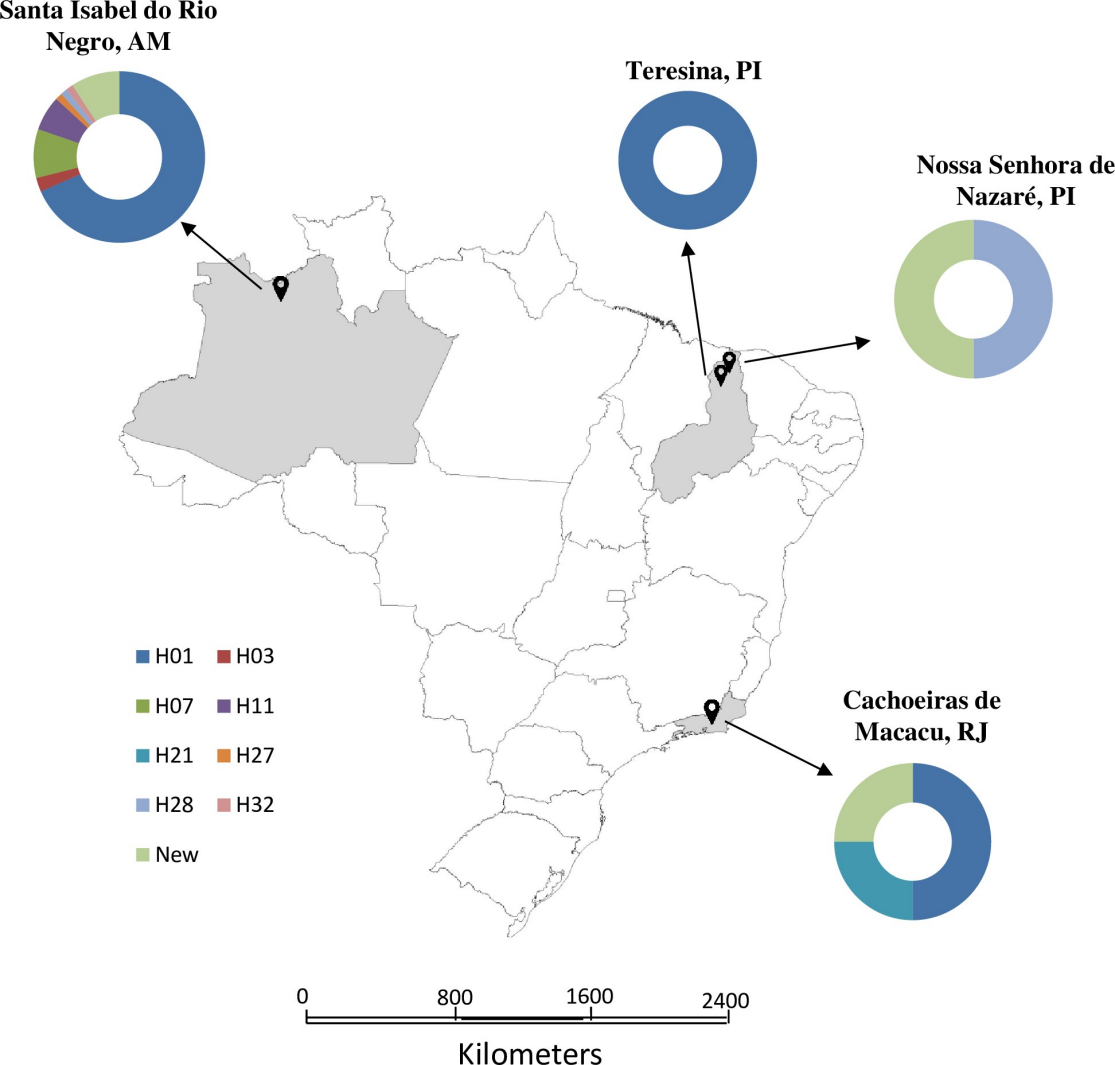
Map of the study areas in Brazil. The haplotypes obtained by the sequencing of *cox*1 locus are shown in the graphs. The map was created using Terraview v.4.2.2 (www.dpi.inpe.br/terraview).

**Fig 3:**
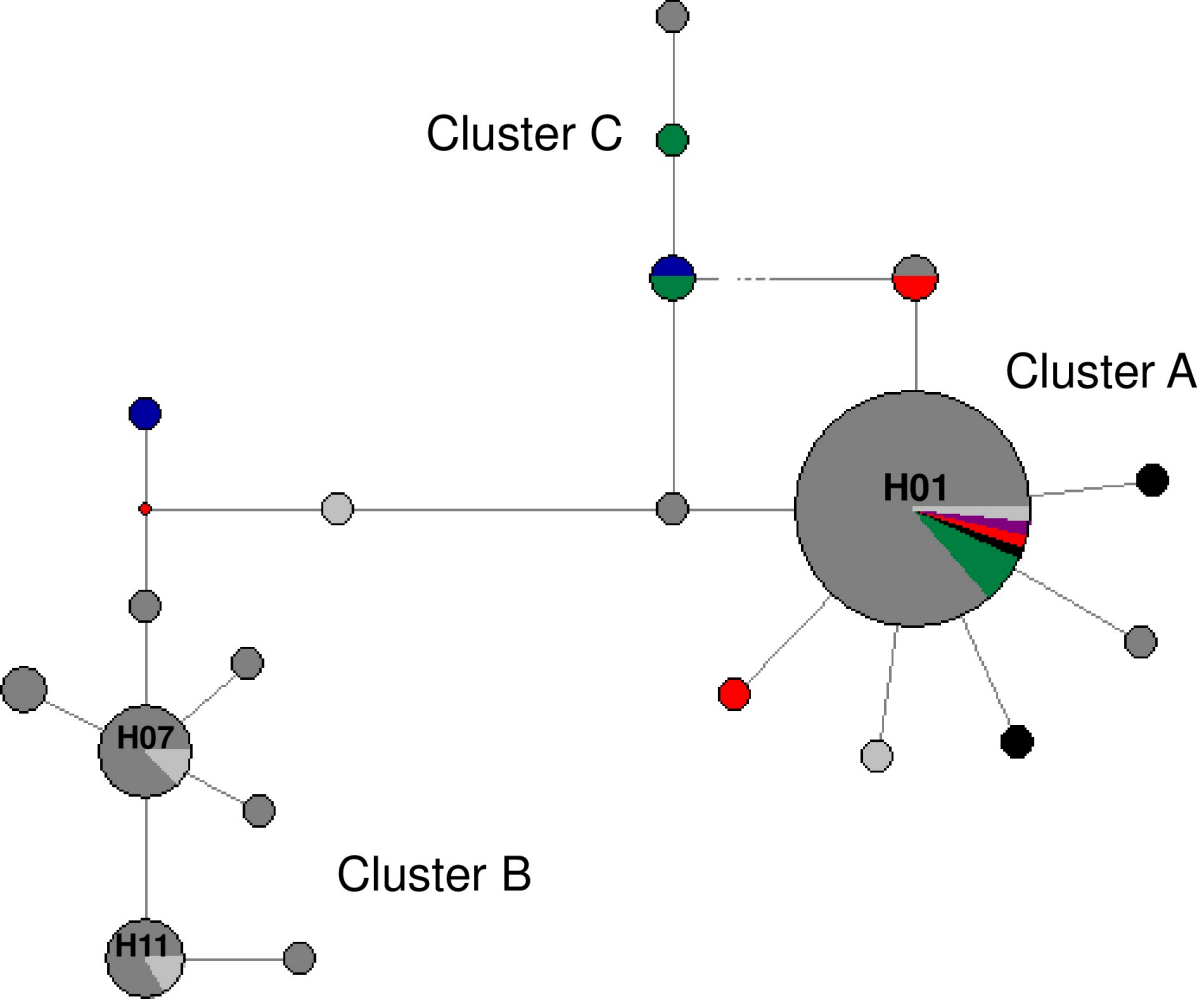
MJ network of 383 bp *cox*1 locus of *Ascaris* spp. from Brazil. The colors of the circles indicate the isolation locality (city). Dark gray: SIRN-AM from this study; light gray: SIRN-AM [21]; green: TER-PI; blue: NSN-PI; red: GUA-RJ [20]; black: CAM-RJ; violet: MG. The area of the circle is proportional to the sequence number.

A MJ network based on *cox*1 locus (383 bp) revealed three main clusters of haplotypes, one centered around haplotypes H01/H28/H32 and the other around haplotype H07/H11 (Fig 1). The haplotype clusters present a star-like shape, as expected from *Ascaris* sp. Seventeen different *cox*1 haplotypes were identified, nine of which were novel. Any amino acid changes in the sequences are described in the supplemental material (S2 Table). Eight of the haplotypes found had already been described (H01, H03, H07, H11, H21, H27, H28, and H32). Table 2 summarizes haplotype abundance by location. The H01 was by far the most abundant haplotype, particularly in humans from SIRN-AM (n = 52). Five haplotypes were rare. In pigs, two new haplotypes were found, as well as the haplotype H28, of human origin. The *cox*1 haplotypes described in this study had a heterogeneous distribution (Fig 2), showing no pattern by geographic region, and high haplotype diversity (general 0.6156 ± 0.0620, Table 2 shows H ± SD by locality). Four haplotypes (H01, H07, H11, and H32) had already been described in Brazil (Fig 3). Pairwise F_st_ analysis comparing Brazilian haplotypes (four localities described in this study and three previously published) indicate significant genetic differentiation (p-value ≤0.05) (Table 3).

**Table 2:** *cox*1 and *nad*1 haplotype abundance and haplotype diversity, by location.

Locus	Haplotype	Locality				Total
		SIRN-AM	NSN-PI	TER-PI	CAM-RJ	
***cox*1** ^**a**^	H01	52	-	6	2	60
	H03	2	-	-	-	2
	H07	7	-	-	-	7
	H11	5	-	-	-	5
	H21	-	-	-	1	1
	H27	1	-	-	-	1
	H28	-	1	1	-	2
	H32	1	-	-	-	1
	New	6	1	1	1	9
	Total	74	2	8	4	88
	H ± SD	0.5082 ± 0.699	1.0 ± 0.5000	0.600 ± 0.2152	1.0 ± 0.2722	0.6156 ± 0.0620
***nad*1** ^**b**^	Previously described	29	3	4	-	36
	New	33	-	1	4	38
	Total	62	3	5	4	74
	H ± SD	0.8772 ± 0.0401	0.6667 ± 0.3143	0.700 ± 0.2184	1.00 ± 0.5000	0.8705 ± 0.0383

^a^ *cox*1 haplotypes H1–H07, H11, and H21-H32 were previously identified in human *Ascaris* from Zanzibar [32], China [25], and Uganda [33], respectively.

^b^ *nad*1 haplotype names were not defined by the authors.

H ± SD: haplotype diversity ± standard deviation.

**Table 3:** Population pairwise F_st_ values based on Brazilian *cox*1 data.

Populations	NSN-PI	TER-PI	SIRN-AM	SIRN-AM^a^	GUA-RJ ^b^	CAM-RJ
**NSN-PI**	0					
**TER-PI**	0.0927	0				
**SIRN-AM 2011**	0.0605	0.3125	0			
**SIRN-AM 2008** ^**a**^	0.5205	0.0664	0.2890	0		
**GUA-RJ** ^**b**^	0.0625	0.4345	0.1943	0.1513	0	
**CAM-RJ**	0.1201	0.1328	0.2353	0.2656	0.7128	0

p-value ≤0.05, 1023 permutations. NSN-PI: Nossa Senhora de Nazaré-Piauí; TER-PI: Teresina-Piauí; SIRN-AM: Santa Isabel do Rio Negro-Amazonas; GUA-RJ: Guapimirim-Rio de Janeiro; CAM-RJ: Cachoeiras de Macacu-Rio de Janeiro.

^a^ sequence data published in [21].

^b^ sequence data published in [20].

The *nad*1 haplotype network was very similar to that of the *cox*1 locus, presenting three main groups of haplotypes (S1 Fig). Thirty-two different *nad*1 haplotypes were identified, 26 of which were novel (Table 2). Any amino acid changes in the sequences are described in the supplemental material (S2 Table). Six haplotypes had already been described. A haplotype found in this study has already been described in *Ascaris* from humans and pigs. In addition, two pig samples presented a haplotype of pig origin and a third pig sample had a haplotype of human origin. The haplotype general diversity was 0.8705 ± 0.0383 (Table 2 shows the H ± SD by locality).

**Fig 4:**
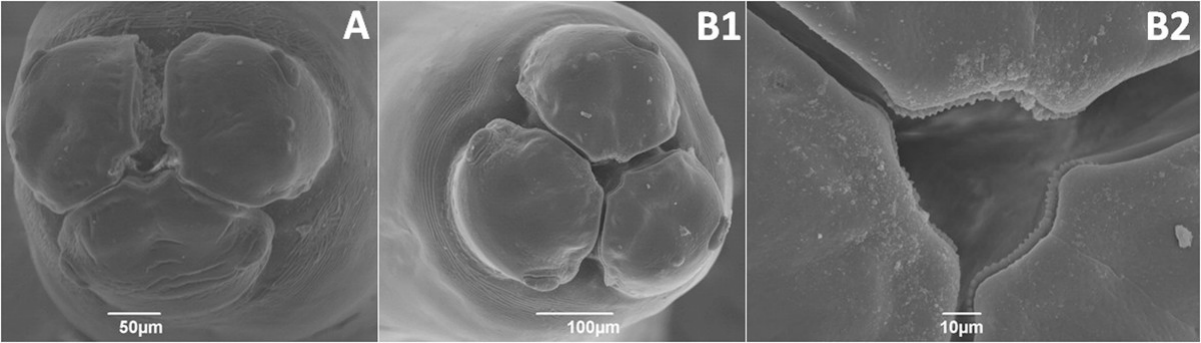
Scanning electron micrographs of the buccal orifice of two adult Ascaris specimens obtained from pig necropsy in the city of Teresina, state of Piauí. A. Six-sided hexagonal shape of the buccal orifice, due to indentations in the three lips. B1. Two lips have indentations and the third is almost straight. B2. At higher magnification, the triangular shape of the denticules is observed.

Scanning electron micrographs were obtained of two specimens, both obtained from pigs (Fig 4). It was observed that the morphology of the buccal orifice was compatible with *A*. *suum*, although the classic hexagonal orifice shape was observed in only one specimen. In one of the specimens it was possible to observe the triangular shape of the denticles, also compatible with *A*. *suum*.

## Discussion

In this study, we describe the genetic diversity of two mitochondrial loci of *Ascaris* sp. from Brazil. The ML tree and MJ network analysis based in both *cox*1 and *nad*1 revealed three main clusters (A-C). Several studies demonstrate this topology for *Ascaris* mitochondrial targets, in which two main groups are formed with i) clusters A and C; and ii) cluster B [34,35]. Our main finding is that, despite this division, there is no separation between specimens obtained from humans and from pigs, presumably *A*. *lumbricoides* and *A*. *suum*. It should, however, be noted that there was relatively low bootstrap support for the internal branches of the dendrograms, which may indicate low phylogenetic resolution when using mitochondrial DNA markers.

We found high haplotype diversity in *A*. *lumbricoides / A*. *suum* samples from Brazil. The number of different *cox*1 haplotypes obtained in this study– 17 haplotypes to *cox*1 and 32 haplotypes to *nad*1 –is similar to that found in previous studies in Asia and Africa [32,35]. Although some localities present low prevalence, these results suggest that there is raised *cox*1 genetic diversity in *Ascaris* across Brazil. The present study adds to the growing database of *Ascaris* sequences from human and pig hosts, since nine and 26 new haplotypes have been described for *cox*1 and *nad*1, respectively. Interestingly, the haplotypes H59, H64, and H73—considered highly divergent haplotypes of *A*. *lumbricoides* / *A*. *suum*–were not found in the studied population. The high haplotypic diversity in Brazil can be explained by the migration for the peopling of the American continent: European settlers and African slaves being introduced from the 17th century and integrated with native Amerindian, which have Asian ancestry [36,37]. During the time of the introduction of other peoples in Brazil, breeding of pigs was established.

Of the eight *cox*1 haplotypes found, four had already been described in the country (H01, H07, H11, and H32) [20,21]. This indicates that, despite the small number of positive cases in some localities, we were able to analyze a number at least reasonably of samples able to reflect the diversity of *Ascaris* in the country. The haplotype H01 was the most common in this study. Interestingly, it has been found in all Brazilian localities studied to date (this study and previous studies by [21] and [20]). This haplotype is widespread in four continents–Asia, Africa, the Americas, and Europe–mainly in human samples [33,35,38]. Furthermore, *Ascaris* sp. isolated from Sumatran orangutans (GenBank accession number LN600399) and chimpanzees [39] from Asia demonstrated genetic similarity with this haplotype. Since it is widely distributed worldwide and has a central position in cluster A, H01 haplotype is considered an ancestor, and to have originated in Africa [39]. The presence of H01 in all Brazilian regions studied can be explained by the migratory phenomena responsible for the peopling of the American continent.

Although all *cox*1 haplotypes (n = 8 previously described) were found to be originally from humans, four of them had already been described as infecting pigs (H01, H07, H28, and H32) [20,21]. Although two samples obtained from pigs in our study (SST2.2 and PBFE3) were classified as the haplotype H28 originally from humans [25], described the same sequence as belonging to the haplotype P1, originally from pigs. Moreover, another pig-origin sequence (SST2.1 sample) differed by only one polymorphism from haplotype H28/P1 and clustered around this haplotype in the network. Another pig sample, PBFE2, situated in cluster B, differed by only three polymorphisms from haplotype H07, and was considered a “pig-like” haplotype by [32]. Therefore, the novel *Ascaris cox*1 haplotypes described here may be new pig-like haplotypes. Nuclear targets have been used in an attempt to unravel the taxonomy of the *Ascaris* genus. The presence of hybrids [40] and possible reproductive isolation between species *A*. *lumbricoides* and *A*. *suum* [41] has been suggested. The presence of shared mitochondrial haplotypes most likely represents retention of ancestral polymorphisms [41].

One limitation of the direct nucleotide sequencing of the amplicon—when it is obtained from egg pool—is that we can lose mixed infections caused by different haplotypes. Despite this, a wide variety of haplotypes were described in this study.

The morphological analysis of two adult specimens obtained by the necropsy of pigs revealed that their mouth parts were compatible with *A*. *suum* species. The nucleotide sequences of the *cox*1 fragment allowed the characterization of these two specimens as *A*. *suum*.

From a One Health perspective, the characterization of the zoonotic potential of *Ascaris lumbricoides* / *Ascaris suum* is important in the improvement of control strategies. In this study, considering the three Brazilian states in which we obtained *Ascaris*-positive samples, the characterization of cross infections was only possible in Piauí, where we examined fecal samples from pigs and humans living in close contact to each other. In NSN-PI, pigs live in close proximity to human dwellings, having free access to houses and streets, so it is to be expected that humans and pigs were infected with the same *Ascaris* haplotype. Intriguingly, in NSN-PI, no human infection was observed with this parasite in the more than 600 analyzed samples [42]. Thus, we could not characterize the zoonotic circulation in communities where the rate of positivity in pigs was high.

It is concluded that *Ascaris* populations obtained from humans and swine in different Brazilian regions are not discriminable through the genetic markers used, which indicates the potential for zoonotic transmission and the need for better control of these infections in swine herds, mainly when created in peridomestic environment.

## Supporting information

S1 Table *Ascaris* spp. reference *cox*1 and *nad*1 sequences used in this study. (PDF)

S2 Table Divergences in amino acids of the *nad*1 and *cox*1 *Ascaris* sequences. (PDF)

S1 Fig ML tree and MJ network of 357 bp *nad*1 locus of *Ascaris* spp. (121 sequences). In ML tree: circle: SIRN-AM; square: TER-PI. Only bootstrap values ≥70% are reported. In MJ network, the colors of the circles indicate the isolation continent: black: America; dark gray: Africa; diagonal: Asia; and light gray: Europe. The area of the circle is proportional to the sequence number.
(TIF)
