# Supplementary material for: Genetic diversity of Ascaris spp. infecting humans and pigs in distinct Brazilian regions, as revealed by mitochondrial DNA
Source: PLoS One. 2019 Jun 24;14(6):e0218867. doi: 10.1371/journal.pone.0218867 (PMC6590885; doi:10.1371/journal.pone.0218867)
Supplement: S1 Table — (PDF) [file pone.0218867.s001.pdf]

S1 Table: *Ascaris* spp. reference *cox*1 and *nad*1 sequences used in this study.

**Species**	**GenBank accession number**	**Country**	**Host**	**Reference**
A. lumbricoides	LN600400	Czech Republic	Pig	[1]
A. suum	KF719134	United Kingdom	Pig	[2]
A. suum	KF719127	Denmark	Pig	[2]
A. suum	KF719142	United Kingdom	Pig	[2]
A. suum	KF719133	Denmark	Pig	[2]
A. suum	KF719132	Uganda	Pig	[2]
A. suum	KF719131	Tanzania	Pig	[2]
Ascaris sp.	KF719144	United Kingdom	Human	[2]
A. lumbricoides	EU582486	Tanzania	Human	[3]
A. lumbricoides	GU326953	Brazil	Human	[4]
A. lumbricoides	GU326952	Brazil	Human	[4]
A. lumbricoides	KF719118	Uganda	Human	[2]
A. lumbricoides	KF719109	Guatemala	Human	[2]
A. lumbricoides	KF536872	Uganda	Human	[5]
A. lumbricoides	KF536871	Uganda	Human	[5]
A. lumbricoides	AJ968332	China	Human	[6]
A. lumbricoides	AJ968331	China	Human	[6]
A. lumbricoides	AJ968330	China	Human	[6]
A. lumbricoides	KM365023	Denmark	Human	[7]
A. lumbricoides	KM365021	Denmark	Human	[7]
A. lumbricoides	KM365020	Denmark	Human	[7]
Ascaris sp.	KY576141	Korea	Human	[8]
Ascaris sp.	KY576140	Korea	Human	[8]
Ascaris sp.	KY576139	Korea	Human	[8]
A. lumbricoides	EU582499	Tanzania	Human	[3]
A. lumbricoides	EU582493	Tanzania	Human	[3]
A. lumbricoides	EU582490	Tanzania	Human	[3]
A. suum	KF719124	Uganda	Pig	[2]
A. suum	AJ968343	China	Pig	[6]
A. suum	AJ968342	China	Pig	[6]
A. suum	AJ968341	China	Pig	[6]
A. suum	AJ968340	China	Pig	[6]
A. suum	AJ968339	China	Pig	[6]
A. suum	AJ968338	China	Pig	[6]
A. suum	KF719138	Uganda	Pig	[2]
Ascaris sp.	KF719145	United Kingdom	Human	[2]
A. suum	AJ968337	China	Pig	[6]
A.lumbricoides	KC839986	China	Chimpanzee	[9]
A.lumbricoides	GU326955	Brazil	Human	[4]
A. lumbricoides	EU582485	Tanzania	Human	[3]

A. lumbricoides	EU582484	Tanzania	Human	[3]
A. lumbricoides	GU326954	Brazil	Human	[4]
A. lumbricoides	GU326950	Angola	Human	[4]
A. lumbricoides	GU326949	Brazil	Human	[4]
A. lumbricoides	GU326948	Brazil	Human	[4]
A. lumbricoides	KF719151	Uganda	Human	[2]
A. lumbricoides	KF719150	Kenya	Human	[2]
A. lumbricoides	KF719149	Uganda	Human	[2]
A. lumbricoides	KF719147	Zambia	Human	[2]
A. lumbricoides	KF719146	Kenya	Human	[2]
A. lumbricoides	KF719117	Uganda	Human	[2]
A. lumbricoides	KF719115	Uganda	Human	[2]
A. lumbricoides	KF719114	Uganda	Human	[2]
A. lumbricoides	KF719113	Uganda	Human	[2]
A. lumbricoides	KF719111	Nepal	Human	[2]
A. lumbricoides	KF719110	Nepal	Human	[2]
A. lumbricoides	KF719108	Bangladesh	Human	[2]
A. lumbricoides	KF719107	Bangladesh	Human	[2]
A. lumbricoides	KF719106	Bangladesh	Human	[2]
A. lumbricoides	KF719104	Bangladesh	Human	[2]
A. lumbricoides	KF719103	Bangladesh	Human	[2]
A. lumbricoides	KF719102	Bangladesh	Human	[2]
A. lumbricoides	KF719094	Bangladesh	Human	[2]
A. lumbricoides	KF536868	Uganda	Human	[5]
A. lumbricoides	KF536867	Uganda	Human	[5]
A. lumbricoides	KF536866	Uganda	Human	[5]
A. lumbricoides	KF536865	Uganda	Human	[5]
A. lumbricoides	KF536864	Uganda	Human	[5]
A. lumbricoides	KF536863	Uganda	Human	[5]
A. lumbricoides	KF536862	Uganda	Human	[5]
A. lumbricoides	KF536861	Uganda	Human	[5]
A. lumbricoides	KF536860	Uganda	Human	[5]
A. lumbricoides	KF536859	Uganda	Human	[5]
A. lumbricoides	AJ968326	China	Human	[6]
A. lumbricoides	AJ968325	China	Human	[6]
A. lumbricoides	KX022398	Brazil	Human	[10]
A. lumbricoides	KX022397	Brazil	Human	[10]
A. lumbricoides	LN600399	Indonesia	Sumatran orangutan	[1]
A. lumbricoides	EU582498	Tanzania	Human	[3]
A. lumbricoides	EU582497	Tanzania	Human	[3]
A. lumbricoides	EU582496	Tanzania	Human	[3]
A. lumbricoides	EU582495	Tanzania	Human	[3]
A. lumbricoides	EU582494	Tanzania	Human	[3]
A. lumbricoides	EU582492	Tanzania	Human	[3]
A. lumbricoides	EU582488	Tanzania	Human	[3]

A. lumbricoides	KF719105	Bangladesh	Human	[2]
A. lumbricoides	EU582487	Tanzania	Human	[3]
A. lumbricoides	KF719112	Nepal	Human	[2]
A. suum	KF719135	Tanzania	Pig	[2]
A. lumbricoides	MF358930	Myanmar	Human	[11]
A. lumbricoides	MF358925	Thailand	Human	[11]
A. lumbricoides	MF358917	Myanmar	Human	[11]
A. lumbricoides	KY045802	?	Human	[12]
A. suum	GU326951	Brazil	Pig	[4]
A. lumbricoides	KF719119	Uganda	Human	[2]
Ascaris sp.	KF719101	Uganda	Pig	[2]
Ascaris sp.	KF719100	Bangladesh	Pig	[2]
Ascaris sp.	KF719099	Tanzania	Pig	[2]
Ascaris sp.	KF719098	Uganda	Pig	[2]
Ascaris sp.	KF719097	Guatemala	Human	[2]
Ascaris sp.	KF719096	Nepal	Human	[2]
Ascaris sp.	KY200860	USA	Pig	[13]
Ascaris sp.	KF719095	Bangladesh	Human	[2]
Ascaris sp.	KY200855	USA	Pig	[13]
A. suum	AJ968336	China	Pig	[6]
A. suum	KF719130	Tanzania	Pig	[2]
A. suum	KF719128	Guatemala	Pig	[2]
A. suum	MF358911	Thailand	Pig	[11]
A. suum	HM602025	Brazil	Pig	[4]
A. lumbricoides	KF719110	Nepal	Human	[2]
A. lumbricoides	MF358934	Myanmar	Human	[11]
A. lumbricoides	MF358918	Thailand	Human	[11]
A. lumbricoides	MF358909	Myanmar	?	[11]
A. lumbricoides	MF358907	Myanmar	Human	[11]
A. lumbricoides	AB591801	Japan	Human	[14]
A. lumbricoides	AJ968333	China	Human	[6]
A. lumbricoides	AJ968329	China	Human	[6]
A. lumbricoides	AJ968328	China	Human	[6]
A. lumbricoides	KF536870	Uganda	Human	[5]
A. lumbricoides	KF719116	Uganda	Human	[2]
A. lumbricoides	KF536869	Uganda	Human	[5]
A. lumbricoides	AJ968327	China	Human	[6]
A. lumbricoides	AJ968324	China	Human	[6]
A. lumbricoides	EU582491	Tanzania	Human	[3]
A. lumbricoides	KF719148	United Kingdom	Human	[2]
A. lumbricoides	EU582489	Tanzania	Human	[2]
A. lumbricoides	MF358926	Thailand	Human	[11]
A. lumbricoides	MF358921	Thailand	Human	[11]
A. lumbricoides	MF358935	Thailand	Human	[11]
A. lumbricoides	MF358919	Myanmar	Human	[11]

A. lumbricoides	MF358915	Thailand	Human	[11]
A. lumbricoides	MF358929	Laos	Human	[11]
A. lumbricoides	MF358923	Laos	Human	[11]
A. lumbricoides	MF358916	Myanmar	Human	[11]
A. lumbricoides	MF358912	Thailand	Human	[11]
A. lumbricoides	MF358910	Laos	Human	[11]
A. lumbricoides	MF358932	Myanmar	Human	[11]
A. suum	KF719137	United Kingdom	Pig	[11]
A. suum	KF719136	Tanzania	Pig	[11]
A. lumbricoides	MF358927	Laos	Human	[11]
A. lumbricoides	MF358924	Thailand	Human	[11]
A. suum	KF719129	Philippines	Pig	[2]
A. suum	KF719126	Denmark	Pig	[2]
A. suum	KF719125	Uganda	Pig	[2]
A. suum	KF719122	Uganda	Pig	[2]
A. suum	HQ704901	China	Pig	[15]
A. suum	KF719141	United Kingdom	Pig	[2]
Ascaris sp.	KF719121	Uganda	Pig	[2]
Ascaris sp.	KF719120	United Kingdom	Human	[2]
A. suum	AJ968335	China	Pig	[6]
A. suum	AJ968334	China	Pig	[6]
A. suum	X54253	?	Pig	[16]
A. suum	MF358933	Thailand	Pig	[11]
A. suum	MF358922	Laos	Pig	[11]
A. suum	KY045800	?	?	[12]
A. suum	MF358931	Thailand	Pig	[11]
A. suum	MF358905	Laos	Pig	[11]
A. suum	AB591802	Japan	Pig	[14]
Ascaris sp.	KF719143	United Kingdom	Human	[2]
Ascaris sp.	KY200853	USA	Pig	[13]
Ascaris sp.	KF719140	Denmark	Pig	[2]
Ascaris sp.	KF719139	Denmark	Human	[2]
Ascaris sp.	KY200852	USA	Pig	[13]
Ascaris sp.	KY200858	USA	Pig	[13]
Ascaris sp.	KY200856	USA	Pig	[13]
Ascaris sp.	KY200857	USA	Pig	[13]
A. lumbricoides	GU326958	Brazil	Human	[4]
A. lumbricoides	GU326957	Brazil	Human	[4]
	GU326956	Brazil	Human	[4]
A. lumbricoides	KX022400	Brazil	Human	[10]
A. lumbricoides	KY045803	?	?	[12]
A. lumbricoides	AP017677	?	?	[12]
Ascaris sp.	KY576136	Korea	Human	[8]
A. lumbricoides	KX022399	Brazil	Human	[10]
A. lumbricoides	GU326963	Brazil	Human	[4]

A. lumbricoides	GU326962	Brazil	Human	[4]
A. lumbricoides	GU326961	Brazil	Human	[4]
A. lumbricoides	GU326960	Brazil	Human	[4]
A. lumbricoides	AJ968354	China	Human	[6]
A. lumbricoides	AJ968353	China	Human	[6]
A. lumbricoides	AJ968352	China	Human	[6]
A. lumbricoides	AJ968351	China	Human	[6]
A. lumbricoides	AJ968350	China	Human	[6]
A. lumbricoides	AJ968349	China	Human	[6]
A. lumbricoides	AJ968348	China	Human	[6]
A. lumbricoides	AJ968347	China	Human	[6]
A. lumbricoides	AJ968346	China	Human	[6]
A. lumbricoides	AJ968345	China	Human	[6]
A. lumbricoides	AJ968344	China	Human	[6]
A. lumbricoides	HQ704900	China	?	[15]
A. lumbricoides	JN801161	Korea	Human	[18]
Ascaris sp.	MH059555	Denmark	Soil	[19]
A. suum	KY045805	?	Pig	[12]
Ascaris sp.	KC839987	China	Gibbon	[9]
Ascaris sp.	KC839986	China	Chimpanzee	[9]
A. suum	KC998843	China	Pig	[20]
A. suum	AJ968355	China	Pig	[6]
A. suum	HQ704901	China	Pig	[15]
A. suum	AJ968366	China	Pig	[6]
A. suum	AJ968357	China	Pig	[6]
A. suum	AJ968359	China	Pig	[6]
A. suum	HM602028	Brazil	Pig	[4]
A. suum	AJ968369	China	Pig	[6]
A. suum	AJ968364	China	Pig	[6]
A. suum	AJ968363	China	Pig	[6]
A. suum	GU326964	Brazil	Pig	[4]
A. suum	HM602026	Brazil	Pig	[4]
A. suum	AJ968368	China	Pig	[6]
A. suum	HM602027	Brazil	Pig	[4]
A. suum	AJ968365	China	Pig	[6]
A. suum	AJ968360	China	Pig	[6]
A. suum	AJ968356	China	Pig	[6]
A. suum	KY045804	?	Pig	[12]
A. suum	AJ968367	China	Pig	[6]
A. suum	AJ968358	China	Pig	[6]

REFERENCES

1. Civanova et al. (unpublished)
2. Betson M, Nejsum P, Bendall RP, Deb RM, Stothard JR. Molecular epidemiology of ascariasis: a global perspective on the transmission dynamics of *Ascaris* in people and pigs. J Infect Dis. 2014 Sep 15;210(6):932-41. doi: 10.1093/infdis/jiu193.
3. Betson M, Halstead FD, Nejsum P, Imison E, Khamis IS, Sousa-Figueiredo JC, Rollinson D, Stothard JR. A molecular epidemiological investigation of *Ascaris* on Unguja, Zanzibar using isoenyzme analysis, DNA barcoding and microsatellite DNA profiling. Trans R Soc Trop Med Hyg. 2011 Jul;105(7):370-9. doi: 10.1016/j.trstmh.2011.04.009.
4. Iniguez AM, Leles D, Jaeger LH, Carvalho-Costa FA, Araújo A; Amazonas Research Group. Genetic characterisation and molecular epidemiology of *Ascaris* spp. from humans and pigs in Brazil. Trans R Soc Trop Med Hyg. 2012 Oct;106(10):604-12. doi: 10.1016/j.trstmh.2012.06.009.
5. Betson M, Nejsum P, Llewellyn-Hughes J, Griffin C, Atuhaire A, Arinaitwe M, Adriko M, Ruggiana A, Turyakira G, Kabatereine NB, Stothard JR. Genetic diversity of *Ascaris* in southwestern Uganda. Trans R Soc Trop Med Hyg. 2012 Feb;106(2):75-83. doi: 10.1016/j.trstmh.2011.10.011.
6. Peng W, Yuan K, Hu M, Zhou X, Gasser RB. Mutation scanning-coupled analysis of haplotypic variability in mitochondrial DNA regions reveals low gene flow between human and porcine *Ascaris* in endemic regions of China. Electrophoresis. 2005. 26(22):4317-26.
7. Søe MJ, Nejsum P, Fredensborg BL, Kapel CMO. DNA Typing of Ancient Parasite Eggs from Environmental Samples Identifies Human and Animal Worm Infections in Viking-Age Settlement. Journal of Parasitology. 2015 101(1):57-63. 2015 https://doi.org/10.1645/14-650.1
8. Hong JH, Oh CS, Seo M, Chai JY, Shin DH. Ancient *Ascaris* DNA Sequences of Cytochrome B, Cytochrome C Oxidase Subunit 1, NADH Dehydrogenase Subunit 1, and Internal Transcribed Spacer 1 Genes from Korean Joseon Mummy Feces. J Parasitol. 2017 Dec;103(6):795-800. doi: 10.1645/16-102. Epub 2017 Jul 24.
9. Xie Y, Niu L, Zhao B, Wang Q, Nong X, Chen L, Zhou X, Gu X, Wang S, Peng X, Yang G. Complete mitochondrial genomes of chimpanzee- and gibbon-derived *Ascaris* isolated from a zoological garden in southwest China. PLoS One. 2013 Dec 17;8(12):e82795. doi: 10.1371/journal.pone.0082795.

10. Da Silva Alves EBS, Conceição MJ, Leles D. *Ascaris lumbricoides*, *Ascaris suum*, or "*Ascaris lumbrisuum*"? J Infect Dis. 2016 Apr 15;213(8):1355. doi: 10.1093/infdis/jiw027. Epub 2016 Feb 4. PubMed PMID: 26908753.
11. Sadaow L, Sanpool O, Phosuk I, Rodpai R, Thanchomnang T, Wijit A, Anamnart W, Laymanivong S, Aung WPP, Janwan P, Maleewong W, Intapan PM. Molecular identification of *Ascaris lumbricoides* and *Ascaris suum* recovered from humans and pigs in Thailand, Lao PDR, and Myanmar. Parasitol Res. 2018 Aug;117(8):2427-2436. doi: 10.1007/s00436-018-5931-6.
12. Nejsum P, Hawash MB, Betson M, Stothard JR, Gasser RB, Andersen LO. *Ascaris* phylogeny based on multiple whole mtDNA genomes. Infect Genet Evol. 2017 Mar;48:4-9. doi: 10.1016/j.meegid.2016.12.003.
13. Jesudoss Chelladurai J, Murphy K, Snobl T, Bader C, West C, Thompson K, Brewer MT. Molecular Epidemiology of *Ascaris* Infection Among Pigs in Iowa. J Infect Dis. 2017 Jan 1;215(1):131-138. doi: 10.1093/infdis/jiw507.
14. Arizono N, Yoshimura Y, Tohzaka N, Yamada M, Tegoshi T, Onishi K, Uchikawa R. Ascariasis in Japan: is pig-derived *Ascaris* infecting humans? Jpn J Infect Dis. 2010 Nov;63(6):447-8.
15. Liu GH, Wu CY, Song HQ, Wei SJ, Xu MJ, Lin RQ, Zhao GH, Huang SY, Zhu XQ. Comparative analyses of the complete mitochondrial genomes of *Ascaris lumbricoides* and *Ascaris suum* from humans and pigs. Gene. 2012. 492(1):110-6. doi: 10.1016/j.gene.2011.10.043.
16. Okimoto R, Macfarlane JL, Wolstenholme DR. Evidence for the frequent use of TTG as the translation initiation codon of mitochondrial protein genes in the nematodes, *Ascaris suum* and *Caenorhabditis elegans*. Nucleic Acids Res. 1990. 18(20):6113-8.
17. Kikuchi et al. (unpublished)
18. Park YC, Kim W, Park JK. The complete mitochondrial genome of human parasitic roundworm, *Ascaris lumbricoides*. Mitochondrial DNA. 2011 Aug;22(4):91-3. doi: 10.3109/19401736.2011.624608.
19. Tams KW, Jensen Søe M, Merkyte I, Valeur Seersholm F, Henriksen PS, Klingenberg S, Willerslev E, Kjær KH, Hansen AJ, Kapel CMO. Parasitic infections and resource economy of Danish Iron Age settlement through ancient DNA sequencing. PLoS One. 2018 Jun 20;13(6):e0197399. doi: 10.1371/journal.pone.0197399.

20. Chang QC, Gao JF, Sheng ZH, Lou Y, Zheng X, Wang CR. Sequence variability in three mitochondrial genes among four roundworm species from wild animals in China. Mitochondrial DNA. 2015 Feb;26(1):75-8. doi: 10.3109/19401736.2013.823171.
