# Supplementary material for: Genetic diversity of Ascaris spp. infecting humans and pigs in distinct Brazilian regions, as revealed by mitochondrial DNA
Source: PLoS One. 2019 Jun 24;14(6):e0218867. doi: 10.1371/journal.pone.0218867 (PMC6590885; doi:10.1371/journal.pone.0218867)
Supplement: S2 Table — (PDF) [file pone.0218867.s002.pdf]

S2 Table: Divergences in amino acids of the *nad*1 and *cox*1 *Ascaris* sequences.

**Isolate**	***nad*1 Amino acids**																		
AP017677 *Ascaris lumbricoides**	W	A	A	H	K	E	G	D	F	F	W	V	Y	W	C	C	E	C	D
A8M3.16.6	.	.	.	Y	K	K	.	.	S	S	R	I	N	.	.	.	.	G	N
DW661.1	.	.	S	.	E	.	.	.	.	.	.	.	.	.	.	.	.	.	.
SAN1065.2	.	.	.	.	E	.	.	.	.	.	.	.	.	.	.	.	.	G	N
DW658.2	.	.	.	.	.	.	.	.	.	.	.	.	.	.	.	.	.	.	N
SAN1035.2	.	.	.	.	K	.	.	.	.	.	.	.	.	.	.	.	.	G	N
SI477.5	.	.	.	.	E	.	.	.	.	.	.	.	.	.	.	.	.	.	N
SAN1010.1	.	.	.	.	.	.	.	.	.	.	.	.	.	.	G	.	.	.	.
SJO645.1	.	.	.	.	E	.	.	.	.	.	.	.	.	.	.	.	.	.	.
DW647.1	.	.	.	.	.	.	.	E	L	.	.	.	.	G	G	.	K	.	N
SAN1046.2	.	.	.	.	E	.	.	.	.	.	.	.	.	G	.	.	K	.	N
SJT251.1	.	.	.	Y	.	K	.	.	S	S	R	I	N	G	G	G	K	G	N
CEN161.1	.	.	.	Y	.	K	.	.	S	S	R	I	N	.	.	.	K	G	.
SI463.1	.	.	.	Y	.	K	.	.	.	S	R	I	N	.	.	.	.	G	.
SI435.3	.	.	.	Y	.	K	.	.	S	S	R	I	N	.	G	.	.	G	.
SI435.1	.	.	.	Y	.	K	.	.	S	S	R	I	N	.	.	.	.	G	.
SI405.2	.	.	.	Y	.	K	.	.	S	S	R	I	N	.	R	R	K	G	N
SAN1003.1	.	R	.	Y	.	K	.	.	S	S	R	I	N	.	.	.	.	G	.
APA801.1	.	.	.	.	.	K	R	.	S	S	.	I	N	.	.	.	.	G	.
SI418.1	.	.	.	Y	.	K	.	.	S	S	R	I	N	G	.	.	.	G	.
SI483.2	.	.	.	Y	.	K	.	.	S	S	R	I	N	G	.	.	K	G	N
SI464.2	.	.	.	Y	.	K	.	.	S	S	R	I	N	.	.	G	.	G	.
SAN1063.4	.	.	.	Y	.	K	.	.	S	S	R	I	N	.	.	.	.	G	.
SJO634.2	.	.	.	Y	.	K	.	.	S	S	R	.	N	.	.	.	K	G	.
SAN1010.2	.	.	.	Y	.	K	.	.	S	S	R	I	N	.	.	.	K	G	N
SI473.3	G	.	.	F	.	K	.	.	S	S	R	I	N	.	.	G	.	G	.
PAP7110.3	.	.	.	Y	.	K	.	.	S	S	R	I	N	.	G	.	K	G	N
**Isolate**	***cox*1 Amino Acids**																		
EU582484 *Ascaris lumbricoides* haplotype 01*	T	M	I	M	S														
SST2.1	.	.	.	I	.														

PBF2	.	.	.	I	R
DW658.2	.	.	.	I	.
SAN1067.4	.	.	M	I	.
SI465.3	.	I	.	I	.
SJO603.1	.	.	.	I	.
SI418.3	.	.	.	I	.
PAP7110.1	P	.	.	.	.
SI418.1	.	.	.	.	.

*Reference sequences. (.): amino acid equal to the reference sequence. A: alanine; C: cysteine; D: aspartic acid; E: glutamic acid; F: phenylalanine; G: glycine; H: histidine; I: isoleucine; K: lysine; M: methionine; N: asparagine; P: proline; R: arginine; S: serine; T: threonine; V: valine; Y: tyrosine; W: tryptophan.
