# Supplementary figures and images for: Genetic diversity of Ascaris spp. infecting humans and pigs in distinct Brazilian regions, as revealed by mitochondrial DNA
Source: PLoS One. 2019 Jun 24;14(6):e0218867. doi: 10.1371/journal.pone.0218867 (PMC6590885; doi:10.1371/journal.pone.0218867)

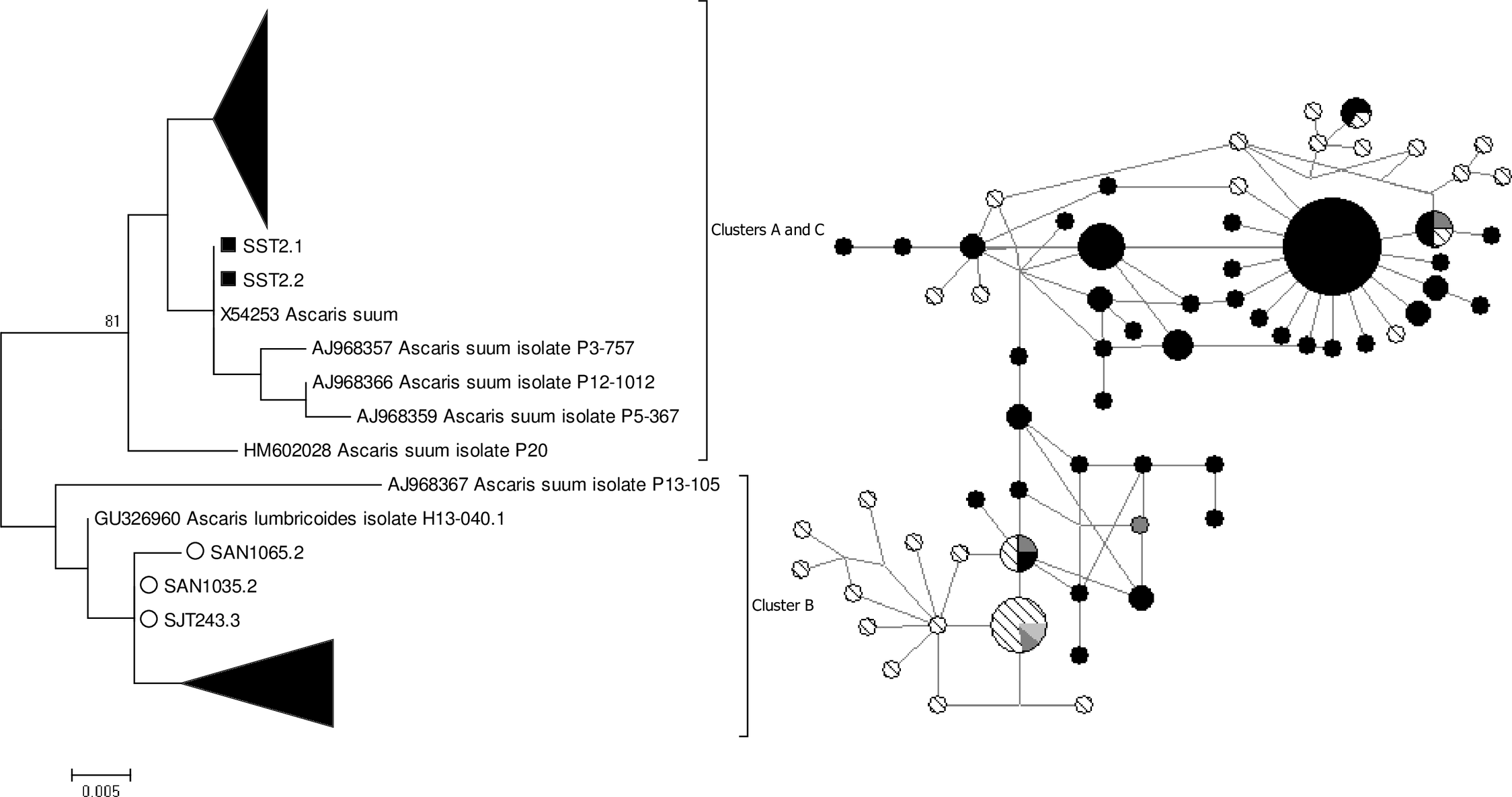

Supplement: S1 Fig — In ML tree: circle: SIRN-AM; square: TER-PI. Only bootstrap values ≥70% are reported. In MJ network, the colors of the circles indicate the isolation continent: black: America; dark gray: Africa; diagonal: Asia; and light gray: Europe. The area of the circle is proportional to the sequence number. (TIF) [file pone.0218867.s003.tif]
